# Mesenchymal Stem Cells as a Gene Delivery Tool: Promise, Problems, and Prospects

**DOI:** 10.3390/pharmaceutics13060843

**Published:** 2021-06-07

**Authors:** Noha Attia, Mohamed Mashal, Gustavo Puras, Jose Luis Pedraz

**Affiliations:** 1 Laboratory of Pharmaceutics, NanoBioCel Research Group, School of Pharmacy, University of the Basque Country (UPV/EHU), Paseo de la Universidad 7, 01006 Vitoria-Gasteiz, Spain; noha.attia@alexmed.edu.eg (N.A.); Mashal313@yahoo.com (M.M.); 2 Department of Basic Sciences, The American University of Antigua-College of Medicine, Coolidge 1451, Antigua and Barbuda; 3 The Center of Research and Evaluation, The American University of Antigua-College of Medicine, Coolidge 1451, Antigua and Barbuda; 4 Histology and Cell Biology Department, Faculty of Medicine, University of Alexandria, Alexandria 21561, Egypt; 5 Networking Research Centre of Bioengineering, Biomaterials and Nanomedicine (CIBER-BBN), Institute of Health Carlos III, 28029 Madrid, Spain; 6 Bioaraba, NanoBioCel Research Group, 01006 Vitoria-Gasteiz, Spain; 7 Laboratory of Pharmacy and Pharmaceutical Technology, School of Pharmacy, University of the Basque Country (UPV/EHU), Paseo de la Universidad 7, 01006 Vitoria-Gasteiz, Spain

**Keywords:** non-viral gene delivery, 3D-bioprinting, transfection, mesenchymal stem cell, cell therapy, gene therapy, niosome, COVID-19

## Abstract

The cell-based approach in gene therapy arises as a promising strategy to provide safe, targeted, and efficient gene delivery. Owing to their unique features, as homing and tumor-tropism, mesenchymal stem cells (MSCs) have recently been introduced as an encouraging vehicle in gene therapy. Nevertheless, non-viral transfer of nucleic acids into MSCs remains limited due to various factors related to the main stakeholders of the process (e.g., nucleic acids, carriers, or cells). In this review, we have summarized the main types of nucleic acids used to transfect MSCs, the pros and cons, and applications of each. Then, we have emphasized on the most efficient lipid-based carriers for nucleic acids to MSCs, their main features, and some of their applications. While a myriad of studies have demonstrated the therapeutic potential for engineered MSCs therapy in various illnesses, optimization for clinical use is an ongoing challenge. On the way of improvement, genetically modified MSCs have been combined with various novel techniques and tools (e.g., exosomes, spheroids, 3D-Bioprinting, etc.,) aiming for more efficient and safe applications in biomedicine.

## 1. MSCs

MSCs is a common acronym used to describe a Mesenchymal Stem Cell, Mesenchymal Stromal Cell, or Medicinal Signaling Cell. However, the debate is still ongoing over which of these long names best describes MSCs [1]. They are an example of “adult” stem cells that could be derived from various tissue types.

### 1.1. MSCs Sources and Features

MSCs have been isolated from almost all tissues [1] and have been reported to play critical roles in many physiological processes, such as tissue homeostasis, immunomodulation, and tissue regeneration [2].

Since the famous publications by Alexander Friedenstein et al., on MSCs, half a century ago, a mounting evidence has been accumulating that bone marrow (BM)-derived MSCs are capable of differentiating into other cells of mesenchymal lineage (e.g., adipocytes, osteoblasts, chondroblasts, myocytes, and tenocytes, etc.,) [3,4]. The authors were able to isolate the plastic-adherent spindle-shaped cells that were capable of self-renewal and showed a multi-differentiation potential.

Later on, more reports unveiled a potential pluripotency where these cells can transdifferentiate into cells of other lineages, endodermal (e.g., muscle, lung, and gut cells, etc.), and ectodermal (e.g., epithelial, and neural cells) [5]. Another interesting feature of MSCs is their homing ability, meaning that they can migrate into injured tissues where they can contribute to the physiological processes in ways more than one. (i) They can differentiate into various local cell types at the injured sites, (ii) they can secrete chemokines, cytokines, and growth factors that help in tissue regeneration, (iii) they can produce non-classical secretory vesicles, known as the extracellular vesicles (EVs), (iv) they can effectively communicate with stressed/injured somatic cells and transfer their cytoplasmic elements and organelles (as mitochondria).

In addition to BM, MSCs can be obtained from various sources such as, adipose connective tissue, synovial fluid, hair follicles, dental pulp, salivary glands, amniotic fluid and membranes, endometrial lining, peripheral and menstrual blood, placenta and fetal membranes, umbilical cord blood, and Wharton’s jelly [6].Therefore, due to the above mentioned appealing features, MSCs have quickly made the transition from benchtop-to-bedside [7].

### 1.2. MSCs Identification

To clearly define MSCs, and develop universal criteria for such cell population, the Mesenchymal and Tissue Stem Cell Committee of the International Society for Cellular Therapy (ISCT) proposed a set of standards for pre-clinical research studies [8].

The minimal criteria of MSCs as determined by the ISCT are the following ones:

The MSCs population must be plastic-adherent when maintained in tissue culture vessels under standard culture conditions.

1. The vast majority of the MSCs population (≥95%) must express the surface markers of CD105, CD73, and CD90. At the same time, they must be negative for CD34, CD45, CD14 or CD11b, CD79a or CD19, and HLA class II.
2. The MSCs population must depict a trilineage differentiation potential to osteoblasts, adipocytes, and chondrocytes.
3. The MSCs population must exhibit immunomodulatory activity (e.g., T-cell suppression via induction of indoleamine 2, 3-dioxygenase (IDO) activity by IFN-γ).

Nevertheless, such historical criteria have not been always correlated with the applicability of these cells in various biomedical purposes. For instance, while CD markers might stay consistent over successive passages, MSCs tend to lose their differentiation or immunomodulatory capabilities [9,10].

Despite the aforementioned criteria, ISCT now suggests a considerable flexibility, particularly when it comes to MSCs the lack of expression of the HLA Class II marker is conditionally expressed once stimulated by specific cytokines.

Therefore, it is crucial to have the process of MSCs characterization well standardized to enable accurate comparison of study outcomes, and to guarantee safety and efficacy in the field. Unfortunately, to date, no single marker has been identified as being exclusively expressed by MSCs [11]. Yet, the number of MSCs markers (positive and negative) is expanding over time to help researchers verifying the MSCs features, thus increasing the confidence in the obtained/transplanted cells.

In addition, various research teams have developed and expanded innovative molecular markers (e.g., proteomic and epigenetic markers, transcriptome analysis, gene signature, etc.,). Despite all these trials to address the thorny question about MSCs identity, there is still little consensus on these characterization methods. Therefore, Arnold I. Caplan [12] has recently suggested the insignificance of characterizing every cell in every MSCs population in vitro. The author believes that most of the propagated MSCs populations have become culture-adapted and can no longer display their innate (in vivo) features, nor their therapeutic behavior, once transplanted.

## 2. Gene Delivery

The Food and Drug Administration (FDA) has defined gene therapy as “the administration of genetic material to modify or manipulate the expression of a gene product or to alter the biological properties of living cells for therapeutic use.” The genetic materials could be transferred into cells in vitro and in vivo. An essential aspect of gene therapy depends on designing a suitable gene delivery system to convey the cargo gene into the target cells. A safe, effective and long-term gene delivery remains an immense challenge for gene therapy. More than half a century after their introduction as a novel therapeutic approach, and despite some adverse effects seen in clinical trials, the concept of gene therapy remains to be acknowledged as a promising therapeutic alternative for various clinical disorders. However, the obstacles encountered have fueled research efforts that led to the improvement of gene carriers in terms of their efficacy and safety profiles. Gene therapy, in its various forms, has depicted marked clinical benefits in patients with countless clinical conditions, such as blindness [13], neuromuscular disease [14], hemophilia [15], cancer [16], etc.

Over the past decades, genetic engineered stem cells were feasibly used in cell-based gene delivery, providing long-term therapeutic effects. Furthermore, the continuous research efforts have been directed toward understanding the behavior of individual stem cells in different tissue microenvironments, in vivo [17]. In parallel, the implementation of more accurate assays for MSCs and enhancement in gene vehicles have increased the gene transfer efficiency. MSCs are genetically modified to produce/overexpress a variety of proteins, in vitro and in vivo, that hold the potential to treat a variety of genetic or acquired diseases, including degenerative disorders [18], or even cancer [19]. The long-term expression ability of such engineered MSCs provides a lifetime therapeutic approach for a myriad of clinical disorders. Nevertheless, quality control of the protocols applied in human gene therapy remains crucial, especially when cells are used as a gene carrier for treatment of hereditary and acquired diseases.

For successful gene delivery to MSCs, the proper choice of the deliverable nucleic acid as well as the delivery carrier/method will determine the transfection outcome. Therefore, in the following section, we will review different types of exogenous nucleic acid cargo along with various non-viral nanocarriers used with MSCs.

### 2.1. Nucleic Acid Therapeutics

Nucleic acids act as drugs aim to treat and/or prevent countless intractable diseases, such as cancer, cardiovascular, neurodegenerative diseases by adding, replacing, editing, or even inhibiting specific target genes or their products [20]. Such therapeutics are based upon a multipurpose platform that has almost unlimited capacity to address unmet clinical needs including vaccination in the current COVID-19 pandemic. Currently, therapeutic nucleic acids could be roughly classified according to their different structures into DNA and RNA drugs. Interestingly, the rapid development in the field of RNA therapeutics has managed to solve the problems of stability, delivery, and immunogenicity. Therefore, various therapeutics were developed and are now commercially available for various diseases (summarized in Table 1).

Despite such achievements, myriad challenges remain to be overcome before their impact on patient’s care is fully understood. In this section, we have discussed some of the most popular nucleic acids used to transfect MSCs, highlighting their advantages and disadvantages (Summarized in Table 2)

#### 2.1.1. Plasmids

Plasmid-based gene therapy was attempted to correct single gene disorders. The first federally approved human gene therapy clinical trial for the treatment of adenosine deaminase deficiency was in 1990. Since then, hundreds of gene therapy protocols have been approved or deployed. On a molecular level, plasmids are circular, double-stranded DNA constructs varying in size from <1000 to >200 000 bp containing transgenes. The main elements in plasmid sequence (e.g., promoters, enhancers, and CpG sites) are known to govern the rate of transgene expression in a cell-dependent context. Therefore, plasmid design can dramatically influence transgene expression [23]. Plasmids can encode different proteins, such as reporter (e.g., *Luciferase*, *EGFP*, etc.,) [24,25] or expression (e.g., *hBMP*, *RPE65*, etc.,) [25,26] genes. Myriad plasmid elements have been studied in the context of MSCs. For instance, pDNA encoding for EGFP and hBMP7 have been successfully transfected into MSCs, resulting in fair transgene expression [25]. Their mechanism of action requires that the plasmid molecules gain access into the nucleus after entering the cytoplasm. Therefore, nuclear localization is a key limiting step that eventually controls the efficiency of gene expression.

#### 2.1.2. Minicircle DNAs

Despite their benefit, the translation of pDNA from benchtop-to-bedside remains highly compromised by their transient expression levels and unwanted backbone elements (bacterial origins of replication and antibiotic resistance). Therefore, minicircle DNAs (mcDNAs) were designed with a minimal backbone to meet the clinical requirements for safe and long-lasting therapeutic transgene expression under the control of mammalian promoters [27,28]. The decreased backbone size was shown to be directly correlated with the levels and extent of transgene expression in mammalian cells [29]. Ji-Young Mun and colleagues had successfully delivered mcDNAs to MSCs by microporation, resulting in an impressive transfection efficiency, in terms of overexpression of C-X-C chemokine receptor type 4 (CXCR4) [30]. They reported the ability of transfected MSCs to efficiently migrate to the injury site in mice. When compared to pDNA, Maria Florian et al., demonstrated that angiopoietin 1 (ANGPT1) encoded in mcDNAs -transfected MSCs could attain a notably higher and prolonged secretion levels of ANGPT1 protein, resulting in superior therapeutic effects animals with acute lung injury [31]. On the other side, Serra J and team reported insignificant difference in transfection results in BM-MSCs with mcDNAs (MC-VEGF) compared to pDNA (pVAX-VEGF) [32]. However, they have highlighted the safety aspect of mcDNAs that come in lower size and have reduced numbers of unmethylated CpG stretches that have been documented to trigger immune response through TLR9 activation. Nonetheless, further studies are still warranted to validate whether TLR9 expression is increased or decreased after transfection when small DNA molecules as mcDNAs are used. Another major concern is the nuclear transport of mcDNAs. Efficient nuclear transport is still required to achieve notable transfection efficiency [29]. Moreover, novel methodologies need to be developed in order to obtain sustainable scale-up production while clinical-grade quality is maintained.

#### 2.1.3. mRNA

MSCs can be transfected more efficiently with mRNAs than with pDNA and mcDNAs, most likely due to abolishing the need for nuclear transport and subsequent transcription. It is noteworthy that mRNA transfection depicts no risk of genome integration. Nevertheless, the protein expression takes place for a shorter duration, which demand repeated transfection. Such transient transfection could be advantageous in certain applications in regenerative medicine [33]. To this end, BM-MSCs were transfected with mRNAs encoding several reprogramming factors (e.g., Oct4, Klf4, Sox2, cMyc, and Lin28) resulting in the formation of iPSC colonies [34]. Moreover, mRNA transfection is being used to simultaneously express multiple proteins such as in the study of Wenbin Liao et al. where MSCs were transfected with mRNA-mediated delivery of PSGL1/SLeX/IL-10 proteins to improve the pathogenesis in mice with experimental autoimmune encephalomyelitis (equivalent to multiple sclerosis in humans) [35]. In general, the simple mRNA delivery approach may serve as an effective means for monitoring the fate of MSCs in vivo to effectively treat a wide range of diseases. The mRNAs-encoding antigens could be utilized as vaccines to provoke the immune system against infectious pathogens, or cancerous cells. Therefore, it is not surprising to know that the first two COVID-19 vaccines, developed by Pfizer-BioNTech and Moderna, that received emergency use authorization by the FDA were mRNA-based. Such breakthrough would not have been possible without critical recent innovations in production of high-quality mRNA as well as the development of safe and efficient materials for in vivo delivery.

#### 2.1.4. Antisense Oligonucleotides and siRNA

In 1978, Zamecnik and colleagues were the first to report the use of synthetic oligonucleotides to silence overexpressed proteins [36]. The antisense oligonucleotides (ASOs) and small interfering RNAs (siRNAs) are the two most widely used strategies for silencing gene expression [37]. The concept is basically to choose a target mRNA of physiological/pathological importance, to which ASOs or siRNAs are manufactured and used to block the translation of this particular mRNA [37]. While the ASO functions as a single strand, siRNA is a double stranded RNA molecule. The passenger strand is lost later, while the remaining guide strand cooperates with RNA-induced silencing complex (RISC) to complementarily bind to target mRNA. Such difference between the two approaches leads to different strengths and weaknesses that affect gene therapy. Based on the oligonucleotides/antisense oligonucleotide technology, Food and Drug Administration (FDA) has approved several drugs summarized in Table 1 (e.g., Pegaptanib, Nusinersen, Golodirsen, etc.,) that were marketed over the past decade for treatment of various diseases. On the other side, only two siRNA drugs, Patisiran and Givosiran were FDA-approved for familial amyloid neuropathies and acute hepatic porphyria, respectively [38].

Recently, Insúa et al. have recently reported the immunostimulatory effect of the PyNTTTTGT ONs on the in vitro and in vivo expansion of MSCs [39]. In addition, they have highlighted the potential clinical application of PyNTTTTGT ONs in tissue repair, either by augmenting the number of MSCs obtained in vitro, or by increasing the quantity of MSCs that are made available in vivo for a natural tissue repair process. Intriguingly, the PyNTTTTGT ONs could be of great benefit if used to complement growth media for MSCs culture in vitro, thus minimizing the addition of fetal calf serum, an arguable component of the culture media. Moreover, the ONs can be used in combination with biomimetic scaffolds to increase the speed of in situ specific tissue development.

Based on their previous findings that knocking down *Smurf1* ubiquitin ligase in rat MSCs would drastically increase bone formation, Patricia García-García and her team have achieved transient *Smurf1* gene silencing in MSCs after single in situ transfection of MSCs with ASOs, resulting in enhanced osteogenesis and mature bone formation in vivo [40].

In terms of cytotoxicity, the ASOs are considered cytotoxic due to their exaggerated pharmacology, resulting from their binding to off-target RNAs, producing undesirable effects. In addition, high doses of ASOs may depict non-antisense effects [41]. In vivo, ASOs administration can induce acute toxicity through activation of the transient complement cascade and the inhibition of the clotting cascade. Another sub chronic toxicity is due to the immunogenicity of ASO, manifested by splenomegaly and lymphoid hyperplasia [42].

Sophie Raisin and her co-workers have efficiently transfected murine BM-MSCs with siRNAs targeting the runt-related transcription factor (Runx2) which is known to be expressed in MSCs and associated with bone differentiation [43]. They provided evidence that the *Runx2* gene was successfully silenced. In another study, E.M. André et al. transfected MSCs using siRNA technology to silence the repressor Element-1 silencing transcription (REST) factor, therefore enhancing the neuronal differentiation. The induced inhibition of REST over time have induced significant neuronal commitment associated with a higher expression of neuronal markers [44].

#### 2.1.5. Aptamers

Aptamers, also known as “synthesized antibody,” are a special group of short single-stranded DNA or RNA oligonucleotides that bind strongly to their target proteins, extracellularly or intracellularly, affecting their functionality. Therefore, they are currently used in several clinical trials [45]. Meng Wang and coworkers developed a novel MSCs aptamer “HM69” based on which, they generated HM69-functionalized nanoparticles (NAB) [46]. Their results demonstrated that aptamer HM69 could recognize and bind to MSCs with high affinity, resulting in the recruitment of endogenous MSCs. Further, the NAB nanoparticles enhanced endogenous stem cell homing and promoted bone defect repair. They concluded that MSCs aptamer HM69 is uniquely attractive for targeted MSCs enrichment. Moreover, the NAB nanoparticles have great potential application as a non-invasive tissue-engineering approach for the treatment of osteochondral lesions.

Recently, Yujian Zou and team selected an aptamer, termed seq3, with high specificity and affinity for mouse BM-MSCs [47]. They developed Seq3-based activatable aptamer probes (AAP) to monitor transplanted BM-MSCs in a mouse model of chronic kidney disease. The AAP have substantially minimized the background signals, thus considered a promising tool for determining the survival status of transplanted BM-MSCs. Nevertheless, aptamers are prone to quick degradation in biological media due to interactions with biomolecules. Many aptamers have been shown to degrade in blood as quickly as a few minutes, which is far too short for most medical applications. Moreover, since most aptamers are relatively small molecules (5 to 15 kDa), they are easily excreted from the body through the kidneys. Interestingly, conjugation of aptamers with large PEG ligands to increase their molecular weight above around 40 kDa could reduce their excretion via the kidneys.

#### 2.1.6. Nucleic Acid Enzymes (Ribozymes and Deoxy Ribozymes)

Ribozymes are another type of NA therapeutic that hold the ability to catalyze the cleavage of phosphodiester bond of specific target RNA transcripts, guided by antisense arms. They eventually regulate gene expression using a catalytic core [48]. In addition to the natural RNA ribozymes, DNAzymes could be obtained through screening technologies, in vitro. The DNAzymes are single-stranded DNA molecules that depict natural catalytic activity. Moreover, they are capable of recruiting small molecules, metal ions, and bacterial pathogens for catalysis. Thereafter, they are used as indicators for these molecules in the diagnosis of certain diseases. Moreover, once coupled with aptamers, they make aptazymes that enable specific detection of a wide variety of signals [49]. Nevertheless, the stability of DNAzymes and ribozymes remains one of the main obstacles that needs to be circumvented before their applications are widely used in treating human diseases. Moreover, other issues as the structure design, delivery, and expression need to be further explored and solved.

In their study, Sophia Millington-Ward and her team investigated a gene therapy for COL1A1-associated dominant-negative forms of OI in BM derived human mesenchymal progenitor cells (MPCs) using a hammerhead ribozyme (Rzpol1a1) [50]. The hammerhead ribozyme has two arms that bind to and cleave the phosphodiester backbone of the mRNA by hydrolysis, thus promoting mRNA degradation and hampering its translation. Therefore, ribozymes have been shown to downregulate COL1A1 transcripts specifically at mutation sites.

Interestingly, DNA enzymes are easier to manufacture compared to their ribozymes counterparts. Moreover, they have less restrictions regarding the cleavage sites, thus supports the choice of targeted cleavers for systematic screening [51]. These advantages, together with their potential in various animal models make DNA enzymes a promising therapeutic agent. However, to the best of our knowledge, they have not been used to modify gene expression in MSCs yet.

#### 2.1.7. MicroRNAs

MicroRNAs (miRNAs) are endogenous non-coding short (20–25 nucleotide) RNA molecules that regulate post-transcriptional gene expression by targeting mRNA for cleavage or translational repression. Overall, miRNAs are tremendously important in controlling cell differentiation and developmental patterning in animals and plants and were found to be implicated in the pathogenesis of many diseases, such as cancers, viral infections, immune diseases, cardiovascular diseases, wound healing, to mention a few. Therefore, miRNA mimics/inhibitors currently in preclinical development have shown potential as novel therapeutic agents. miRNAs are considered critical regulators of the differentiation of BM-MSCs. The two key components in the biogenesis of canonical miRNAs, silencing Dicer or Drosha, block both adipogenic and osteogenic differentiation of BM-MSCs [9]. In their study, Yueqiu Chen and team have achieved transfection of rat BM-MSCs with the agomir or antagomir of miR-133 [52]. Subsequently, they documented significant decrease in hypoxia-induced apoptosis of cells transfected with miR-133 agomir, while the apoptosis rate increased in MSCs transfected with miR-133 antagomir. More importantly, once those miR-133 overexpressing MSCs were transplanted into rats with acute myocardial infarction, a significant improvement in cardiac function was reported compared to their control animals. Further studies indicated that both the inflammation level and the infarct size decreased in miR-133-MSC-injected rat hearts.

In a thought-provoking approach, J. Carthew et al. have developed a simple system for in situ transfection of miRNA and demonstrated its convenience to boost osteogenic differentiation of MSCs within a soft hydrogel scaffold [53]. The addition of PEI-complexed miR-100–5p and miR-143–3p maintained a steady and gradual availability of target miRNAs to the encapsulated cells, more effectively than encapsulating pre-transfected MSCs. This study opens the door for further tailoring the diffusion rate/timing for or miRNAs diffusion.

#### 2.1.8. Short Hairpin RNA (shRNA)

Shortly after the cellular mechanism of RNAi was first described, research on such powerful technique has popped up. This included designing better methods for the successful delivery of shRNAs into mammalian cells. Once inside the cells, Dicer converts the hairpin structure of shRNA into siRNA that ultimately utilizes the RISC mechanism to induce gene suppression. In addition to their fewer off-target effects, shRNAs are advantageous over siRNAs due to the following: (a) Their ability to use viral vectors for delivery to overcome the difficulty of transfecting certain cell types; (b) the possibility to control shRNA expression through inducible or tissue specific promoters; (c) the ability to co-express them with a reporter gene.

In their study, Yingwei Zhu and colleagues have transduced MSCs with shRNA inhibiting the expression of metalloproteinase 1 (TIMP-1) [54]. They investigated the therapeutic effect of TIMP-1-silencing MSCs in a rat model of CCl_4_-induced liver fibrosis when transplanted intravenously. They indicated that transplantation of MSCs expressing TIMP-1-shRNA could inhibit the progression of liver fibrosis and possibly restore the liver function. In another work, Natasha Baker and her coworkers have reported that shRNA-mediated knockdown of caveolin-1 expression enhances MSCs proliferation and osteogenic differentiation [55]. To establish a stable knockdown, they infected human BM-MSCs with a lentiviral construct harboring shRNA targeted against caveolin-1.

In a different approach, Yoon and team achieved a knockdown for nuclear factor erythroid-derived 2-like 2 (NRF2) in early passage MSCs using two different shRNAs (shRNA-1 or shRNA2) [56]. Such regulation of gene expression helped them studying the role of NRF2 in protecting MSCs against cell death and apoptosis caused by oxidative stress and retaining their multi-differentiation potential.

However, nucleic acids are believed to hold the ability to manipulate cellular functions, leading to the development of specific and safe novel medicines, it is noteworthy to highlight that nucleic acids still have several drawbacks for their development as drugs. Their chemical modifications have significantly boosted their specificity and stability properties against nucleases, without losing their biological properties. Nevertheless, the delivery of most of nucleic acids remains as a major hurdle toward the development of nucleic acids as therapeutic agents. Therefore, in the next section we discuss the nucleic acid delivery carriers to MSCs with much emphasis on the lipid-based carriers.

### 2.2. Nucleic Acid Delivery Carriers

**Figure 1:**
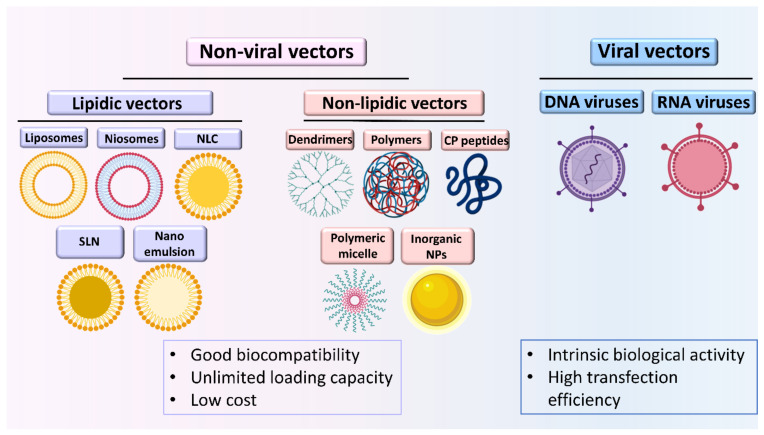
Viral and non-viral carriers of nucleic acids. NLC: nanostructured lipid carrier, SLN: solid lipid nanoparticles, CP: cell penetrating, NPs: nanoparticles.

Different from traditional therapeutics, the cellular internalization “endocytosis” mechanism is a major limiting step for nucleic acids. Therefore, the need for delivery vectors to transport them to the cell nucleus/cytosol is crucial [61,62]. Traditionally, there are two main categories for gene delivery carries, namely the viral and non-viral ones (illustrated in Figure 1). The viral gene carrier, such as adenovirus, retrovirus, and lentivirus, are used to deliver genes into cells via the process of transduction. Despite the high transduction efficiency and stable gene expression that viral vectors tend to provide, myriad challenges remain to be circumvented (e.g., immunogenicity, mutagenesis, limited DNA loading capacity, taxing large-scale production, etc.). Interestingly, non-viral gene carriers, including lipid-based DNA therapeutics, are considered promising alternatives to their viral counterparts as they hold the potential to address most of such limitations [63,64]. Moreover, the transient gene expression provided by most of these systems is compatible with normal tissue repair. Currently, several non-viral tools are commonly used for gene delivery, such as lipids nanoparticles NPs (e.g., solid lipid NPs, liposomes, niosomes, etc.,), polymers (e.g., poly (β-amino ester)s [60], heparin and chitosan-based NPs, etc.,) [65], and inorganic NPs (e.g., superparamagnetic iron oxide, silica NPs, quantum dots, gold NPs, carbon nanotubes, calcium phosphate NPs, etc.,). While such systems can be used to transfect MSCs with various nucleic acid, DNA and RNA, cargo, the development of an optimal non-viral vector to transfect such valuable cells remain a challenging goal.

Lipid-based gene delivery systems became one of the first non-viral gene delivery systems to be approved by the FDA and other regulatory agencies [66]. Lipid nanoparticles (LNP) are interesting drug/gene delivery systems for many reasons. For instance, they can carry both lipophilic and hydrophilic categories of drugs to specific cells/tissues without being toxic, they are also famous for their good biocompatibility and biodegradability. Therefore, in this section, we will emphasis some of their merits and applications.

#### 2.2.1. Lipid-Based Genetic Carrier

##### Solid Lipid Nanoparticles (SLNs)

Solid lipid nanoparticles (SLNs), formerly known as lipospheres, are one of the pharmaceutical nanocarriers that hold great promise for controlled drug delivery. SLNs are typically composed of biodegradable and safe lipidic components [67]. They can carry a variety of therapeutics, such as small drug molecules, large biomacromolecules (polysaccharides, etc.,), genetic material, in addition to the vaccine antigens due to their cationic lipid content that provides a positive surface potential favoring the binding to nucleic acids such as like pDNA, siRNA, miRNA [68].

Chongrui Jin et al. have recently constructed nanosphere-small MyoD activating RNA-bladder acellular matrix graft scaffold loaded with adipose-derived stem cells (ADSC) to explore its effect on smooth muscle regeneration and bladder repair function in rats [69]. They have found that saRNA-MyoD lipid NPs promote a sustained release of MyoD and smooth muscle regeneration with scaffold degradation in rats. The bonding of saMyoD to the lipid NPs have provided a targeted sustained release system, evading undesired off target effects. Therefore, this method could reduce the potential risk of free diffusion of cytokines in vivo, thus promoting their efficient utilization.

##### Liposomes

**Figure 2:**
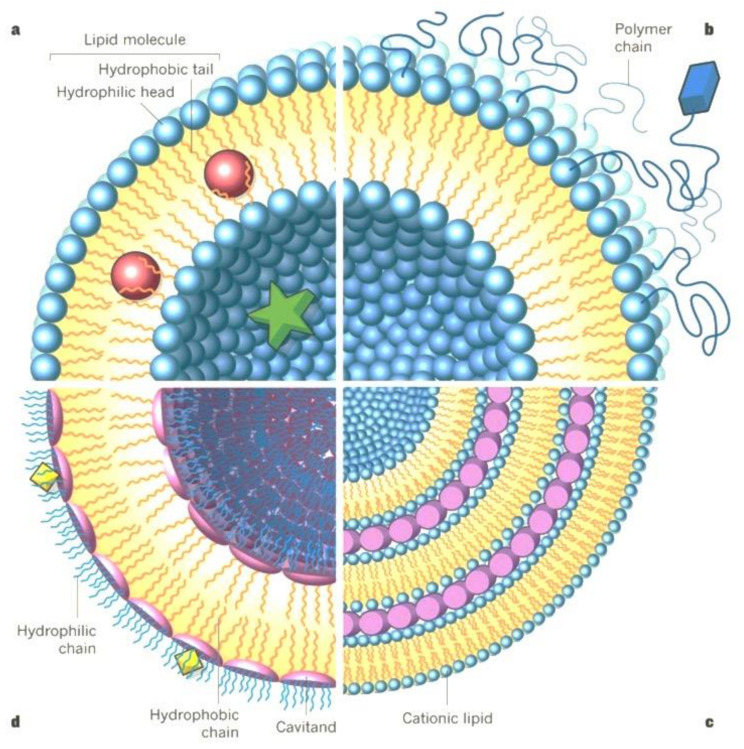
Schematic structure of liposomes showing different compositions. [70]. CC BY 4.0 license.

Liposomes are spherical vesicles having an aqueous core enclosed by one or more phospholipid bilayers or lamellae. They are mainly composed of several structural components such as, phospholipids and cholesterol. Liposomes are assembled as one or more simple or functional concentric lipid bilayer membranes that enclose hydrophilic spaces (Figure 2).

Over the years, different methodologies for large scale liposome preparation have been developed and optimized recently by including various synthetic modifications to the phospholipid component to enhance tissue-specific targeting and evading detection and clearance by the immune system. Therefore, several commercially available liposomes (e.g., Lipofectamine^®^ 2000, TransFast^®^, ScreenFect^®^, Lipofectin 2100^®^, etc.,) are widely used for gene delivery purposes in vitro, in vivo. Moreover, liposome-based products are used in patients enrolled to clinical trials, up to phase IV [71]. Recently, Qiao et al. have used one of the commercially available liposomes (Lipofectamine 2000) to transfect BM-MSCs with miR-203 mimics and miR203 inhibitor [72]. They found that overexpression of miR-203 increased the level of osteogenic related genes but decreased that of adipogenic related genes, while knockdown of miR-203 led to the opposite results. Similarly, the same liposome transfection reagent has been used as a positive control in several transfection experiments on MSCs as well as other cell lines [25,61,62,64,73]. Nonetheless, the success of lipofection is controlled mainly by their mechanical properties, route of administration and delivery barriers (e.g., in vivo stability, permeation of particles through tissues, low target cell specificity, reticuloendothelial system (RES), subcellular localization, etc.) that they must overcome to ultimately preform their function.

##### Niosomes

Niosomes are membranous vesicles composed mainly of non-ionic surfactants, amphipathic compounds with a net neutral charge. The non-ionic surfactant component of niosomes tend to be oriented in a way that hydrophilic ends face the aqueous phase (outward), whereas the hydrophobic ends face inward to form a closed bilayer structure that could enclose solutes in the aqueous solution. Consequently, the closed bilayer structure of niosomes has hydrophilic inner and outer surfaces, with a sandwiched lipophilic area in between. The non-ionic surfactants are safe and affordable for the use in biomedicine as carriers for genes or hydrophilic/hydrophobic drugs [74]. Niosomes are prepared by various methods, such as ether injection, sonication, and microfluidics, to mention a few [75].

Owing to their structural similarities with liposomes, niosomes were proposed for targeted drug delivery by promoting sustained drug release into specific cells and tissues. Moreover, their efficacy and safety have been proven in numerous pre-clinical and clinical studies that emphasized the biocompatibility, biodegradability, and low immunogenicity of niosome components (i.e., non-ionic surfactants, cholesterol, fatty acids, charged molecules, etc.,) [76]. Cytotoxicity is a major factor that should not be overlooked when niosomes are used as a therapeutic tool. Niosomes are way more tolerated by transfected cells compared to their anionic or cationic counterparts [77]. Therefore, the combination of relatively cytotoxic cationic lipids with non-ionic molecules to formulate niosomes could reduce such undesirable toxicity [78]. In addition to the aforementioned properties, the low cost and easy preparation of niosomes have paved their way to stand out versus liposomes (as summarized in Table 3) and other conventional systems for drug/gene delivery.

In a recent study, MSCs line has been engineered to overexpress hBMP7 by transfecting the hBMP7 gene using the pUNO1-hBMP-7 plasmids and novel cationic niosomes. Two days following transfection, cells secreted up to 1460 pg of hBMP7 protein per ml [25]. Such amount of overexpressed hBMP7 protein was sufficient to induce spontaneous osteogenesis in vitro. Therefore, we suggested that BM-MSCs overexpressing hBMP-7 could be considered as a reliable delivery tool of hBMP-7 to target tissues. In addition, they are still considered as proliferating and bone-forming cells to target sites where bone regeneration is needed.

## 3. Applications of Engineered MSCs

As mentioned above, there are various approaches through which genetically modified MSCs can be applied to achieve therapeutic impact in different clinical conditions. MSCs were used to deliver a myriad of growth factors [80,81], cytokines [82], transcription factors [43], or even suicide gene [83,84] with various potential clinical purposes. Some of these applications are reviewed next and summarized in Table 4.

### 3.1. Bone and Cartilage

MSCs are well-known of their natural ability to differentiate into cells with bone and cartilage phenotypes. Moreover, they are reported to induce a paracrine effect that boosts the natural repair process in case of injury. This latter mechanism was the basis for most of the studies using MSCs to deliver/overexpress various types of factors known to be key in repair mechanisms, thus being therapeutically useful. Among those factors were the BMPs, BMP2 [85,86,87], and BMP7 [25]. BMPs are known for their ability to enhance bone regeneration through various mechanisms, such as signaling the chemotaxis, in addition to proliferation, and differentiation of osteoprogenitor cells. As a pro-angiogenic growth factor, MSCs overexpressing VEGF was also used to improve the vascularity of microenvironment [88]. Other mechanisms by which engineered MSCs boosted bone regeneration was to transduce transcription factors as Trb3 [89] or miRNAs (as miR-5106) [90] to manipulate cell differentiation, or to transiently silence the Smurf1 gene [40].

### 3.2. Central Nervous System

Many neurological disorders are difficult to treat due to the high sensitivity of neurons to the hostile environment following the injury/pathology. In addition, to the doubtful regenerative capacity in the first place. Moreover, the blood-brain barrier (BBB), as a tough gatekeeper, makes it difficult to treat the diseases of the CNS, including cancer. However, owing to their ability to cross the BBB [91], MSCs are proposed as a useful carrier of genes/drugs to the CNS. For instance, they can be used to deliver growth factors as hepatocyte growth factor (HGF) or fibroblast growth factor (FGF) 21, to improve repair after a spinal cord [80] or brain injury [92], respectively. Although cerebral ischemia is considered a major clinical problem worldwide, the treatment available is quite limited. In that regard, MSCs used to deliver genes as HGF [81], IL-10 [82], VEGF [93],or CXCR4 [94] were found to boost neovascularization and enhance nerve tissue regeneration in animal models of cerebral ischemia.

### 3.3. Myocardial Ischemia

Ischemic heart disease is a leading cause of death worldwide. Improvement of blood flow to the heart muscle is considered a crucial treatment option. As mentioned above with CNS, MSCs could be quite helpful to improve the microvasculature of the heart muscle through delivering key growth factors, such as VEGF [95,96] and HGF [97]. The activated serine/threonine kinase AKT, as the major substrate to phosphoinositide 3-kinase (PI3K), is reported to control various cellular functions (e.g., cell proliferation, metabolism, and survival, etc.,), in addition to being able to boost angiogenesis. The AKT, successfully delivered to the myocardium via the AKT-overexpressing MSCs, was reported to induce a dramatic early reduction in the infarct size and improve the cardiac function [98]. In another approach to activate the AKT/ERK-related pathway, MSCs overexpressing miR-126 were able to boosted angiogenesis and improved cardiac function in the infarcted area of the myocardium of mice [99].

### 3.4. Cancer

Following cardiovascular disorders, cancer ranks the second most common cause of death in many countries. Each year, half of the tens of millions of patients diagnosed with cancer eventually die from it. Over the past two decades, there have been various trials to use genetically modified MSCs a possible therapeutic approach. One of such attempts was to overexpress the suicide gene thymidine kinase (TK) either alone [83,84], combined with valproic acid (VPA) [100], or with tumor necrosis factor-related apoptosis-inducing ligand (TRAIL) [101] to promote cancer cell death, thus regressing tumor growth. Other studies used a different strategy depending on the overexpression of antitumor cytokines, as IL7 [102] and IFN-β [103]. Moreover, various approaches were reported to combat tumor vascularity using genetically modified cells that overexpress anti-angiogenesis molecules, such as Endostatin [104] and kringle1-5 protein [105]. Another recent study used MSCs as vehicles to deliver immuno-modulatory proteins, IL7 and IL12, to improve the anti-tumor impact of CAR T cells in a transplant tumor model [102].

### 3.5. COVID-19

In quick response to the COVID-19 pandemic, MSCs are being utilized in several clinical trials studying SARS-COV2-induced pneumonia [106]. For instance, Zikuan Leng et al. have recently reported that the transplantation of MSCs, that do not express ACE2 and TMPRSS2, had improved pulmonary function among other parameters in COVID-19 patients [107]. Owing to the unique immunomodulatory capacity of MSCs, the serum levels of pro-inflammatory cytokines and chemokines were dramatically lowered in patients after intravenous transplantation of MSCs. Moreover, the increased IL-10 and VEGF were behind the improved repair of lung tissue. In general, acute respiratory distress syndrome (ARDS) results in increased pulmonary inflammation and vascular permeability causing high mortality in critically ill patients. MSCs were found to reduce such abnormalities through the secretion of paracrine/endocrine factors and/or direct interaction with host immune cells. Moreover, when MSCs were transfected to overexpress the angiopoietin-1 protein, a vascular protective factor, pulmonary endothelial permeability was notably reduced in mice with ARDS.

The alpha-1 antitrypsin (AAT) protein is acknowledged for being one of the key players in the acute phase anti-inflammatory response [108]. Therefore, MSCs engineering appears to be a promising strategy to overexpress the AAT protein, not only to treat AAT deficiency, but also to augment AAT in patients with normal plasma levels of AAT [109]. This approach is quite flexible as patients would benefit from the versatile secretome of MSCs in addition to the expression of AAT protein [110]. If fact, several gene delivery trials were conducted using various virus carriers. Nevertheless, results obtained, to date, did not achieve protective levels of AAT protein in the target lung tissue [111]. Therefore, MSC-based gene therapy could be a promising therapeutic tactic to circumvent the problem of targeted delivery and satisfactory level of gene expression [1], especially when we know that most of the intravenously administered MSCs seem to be trapped in the lungs during the first pass [112].

### 3.6. Other Applications

Various other clinical conditions could benefit from MSCs as a gene delivery carrier. For instance, human telomerase reverse transcriptase enzyme (hTERT)-transfected MSCs could differentiate into islet-like cells that were found to reverse hyperglycemia in diabetic mouse model [113]. Moreover, in complicated skin wounds [114], the gene delivery system could be used as a multifaceted approach to enhance the healing process via VEGF-induced angiogenesis, hand in hand with combating bacterial infection.

## 4. Future Perspectives

**Figure 3:**
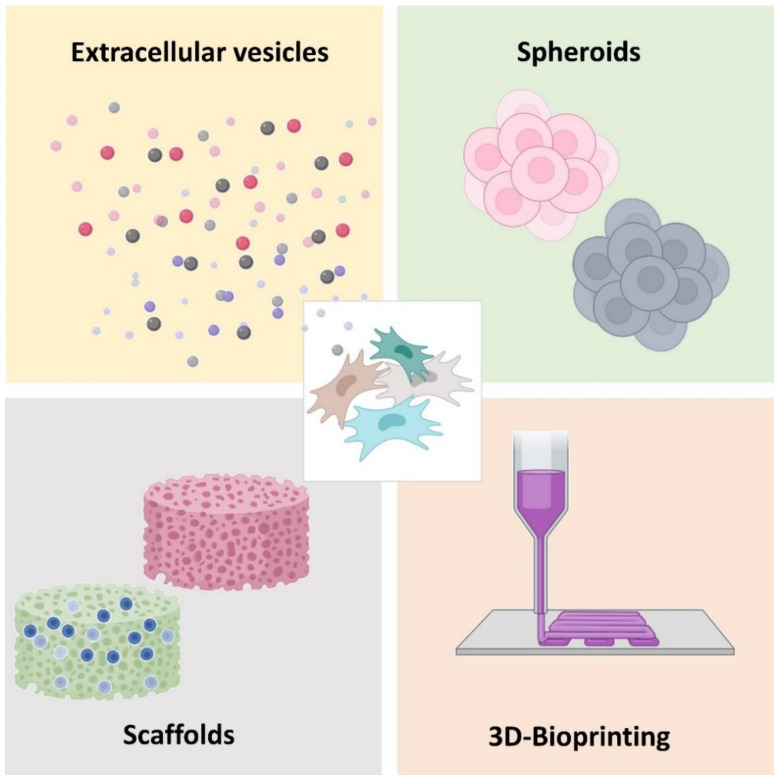
Some innovative approaches for improving gene delivery by MSCs.

In addition to all of the abovementioned considerations about the MSCs population, the other stakeholders of gene delivery (e.g., nucleic acids, carriers, and transfection technique) should be optimized in a holistic approach for better transfection of MSCs. However, the transfection efficiency remains a fundamental limitation for applications that make use of non-viral gene transfer to MSCs, therefore innovative approaches (summarized in Figure 3) for improving non-viral gene delivery are being investigated, as described next.

### 4.1. Exosomes

As mentioned before, the pleiotropic effects of MSCs are now believed to be mediated mainly by the paracrine secretion of various molecules [6]. Exosomes are nanoscale extracellular vesicles that are considered an important class of such paracrine mediators. Myriad studies have reported that exosomes were behind the therapeutic impact of MSCs in various experimental models. Therefore, exosomes were among the main pillars of the novel cell-free therapeutic approach for treatment of a variety of disorders [122]. Moreover, exosomes hold a great potential as drug delivery vehicles due to their natural properties (e.g., cargo molecules, intrinsic long-term circulatory capability, excellent biocompatibility, etc.,) [123] rendering them ideal carrier for a variety of proteins and nucleic acids. Nevertheless, there is still an unmet need to develop an effective method for loading specific proteins/nucleic acids into exosomes [124].

In their recent work, Ran Kim et al. have injected U87 glioma cells into mice exposed to exosomes derived from miRNA-584-5p-transfected MSCs, to confirm the influence of exosome miRNA on the progression of glioma [125]. Their results were encouraging enough to recommend exosomes derived from miRNA-transfected MSCs as a new targeted cancer therapy. In another study, silencing of IL 10 (IL10 knock-down) was done by Alfonso Eirin et al. to adipose tissue MSCs (via siRNA) from which EVs were obtained to investigate the particular renoprotective role of the IL10 content of EVs [126]. Similar to the effect of AT-MSCs, their EVs were found to attenuate renal fibrosis, ultimately improving stenotic kidney function. Notably, the beneficial effects of EVs were blunted in pigs that received IL-10-silenced EVs, supporting an important contribution of this cytokine to several aspects of renal function. In this way, engineered EVs could be obtained and used in cell-free therapy.

Another interesting approach is the exogenous loading of EVs, after their isolation, through various methods, such as electroporation, simple incubation, chemical transfection, or others. For instance, chemical transfection methods have been utilized to load siRNA into EVs by simply incubating exosomes with siRNA-lipofectamine 2000 complexes (siRNA embedded in lipid micelles). However, further studies are still needed to enhance the transfection efficiency and to discard the excess transfection reagent.

### 4.2. Spheroids

MSCs can be used to treat osteoarthritis, yet the therapy is hindered by massive MSCs death after intra-articular implantation. Furthermore, MSCs barely secrete IL-4, a potent anti-inflammatory cytokine. Seuk Young Song et al. suggested a combination of both Il-4 gene transfection (using GFP-expressing plasmids and a cationic liposome-based reagent) and spheroid formation to potentiate the therapeutic efficacy of MSCs for osteoarthritis [127]. MSCs in spheroids depicted better survival compared to naïve MSCs in vitro and in vivo. Despite the low secretion of IL-4 protein (7 ng mL^−1^/12 h), transfected MSCs spheroids induced significantly more cartilage protection and pain relief than conventional naïve MSCs.

### 4.3. Scaffolds

In general, 3D-scaffolds represent a promising modality in promoting tissue regeneration and treating diseases. In general, the 3D environments influence integrin-based interaction between MSCs and the surrounding extracellular matrix, resulting in modulation of cell signaling, that would eventually regulate gene expression [128]. Therefore, significant attention was directed toward exploring the potential of combining non-viral vectors, as liposomes, to design vector-scaffold composite systems for various biomedical applications. Liposome-loaded scaffolds are designed mainly to combine the advantages of both liposomes’ biocompatibility and scaffolds’ strength, enhancing their applications in in-tissue regeneration for bone, teeth, spinal cord, and skin disorders.

In a recent study, Li-Ming Li and colleagues applied a three-dimensional gene transfection technology by transfecting MSCs with brain derived neurotrophic factor (BDNF) gene using the liposomal gene carrier, ScreenFect^®^ A [129]. To improve gene expression as well as cellular survival after in vivo implantation of MSCs, an adhesive peptide (PPFLMLLKGSTR)-modified hydrogel scaffold was constructed using hyaluronic acid. Compared to untransfected MSCs, the transfected cells exhibited sustained gene expression and prolonged survival in the 3D-scaffold in vitro, and improved spinal tissue integrity, inhibited glial scar formation, and alleviated inflammatory response in vivo.

In another study, Jiabing Fan et al. reported that Tribbles homolog 3, an intracellular kinase-like molecule that modifies cellular survival and metabolism, does serve as a key molecular switch controlling adipocyte-osteoblast differentiation of MSCs, in addition to regulating MSCs lineage fate through the BMP and Wnt signaling [89]. They employed scaffold-mediated local Trb3 transfer (plasmid-expressed Trb3 using Lipofectamine^®^ 2000 transfection of MSCs) to improve bone healing in a clinically related mandibular bone defect model.

**Figure 4:**
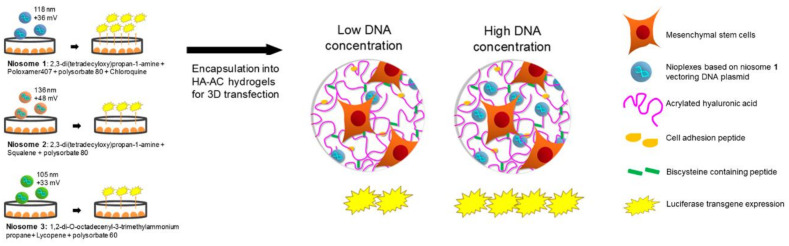
Nioplexes-loaded HA hydrogels for transfection of MSCs [130]. CC BY 4.0 license.

In a similar context, our group has evaluated a new combination of niosomes, non-viral pDNA carrier, and hyaluronic acid HA hydrogel scaffolds [130]. Both components are widely studied owing to their safe and biocompatible profile. We evaluated different types of niosomes varying in their cationic lipid, helper lipid, and non-ionic surfactant composition. The ability of pDNA-niosome complex-loaded HA hydrogels was found to hold little or no particle aggregation, allow for extensive cell spreading, and to efficiently transfect encapsulated mMSCs with minimal cytotoxicity. Such in vitro model could be utilized to design novel and effective platforms for in vivo local non-viral gene delivery applications (Figure 4).

### 4.4. D-Bioprinting

**Figure 5:**
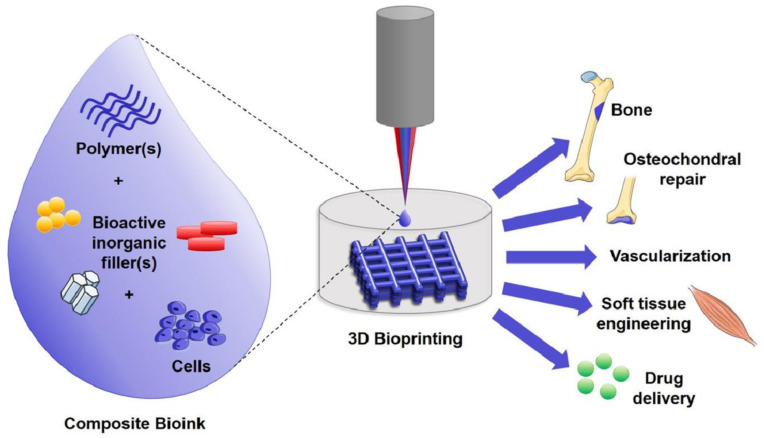
Multi-material bioinks used to bioprint 3D constructs for various biological applications [131].CC BY-NC-ND 4.0.

Tissue engineering of complex solid organs has been such a challenging approach which led to the development of bioprinting as a game changer technology holding great promise for versatile biomedical applications. In general, bioprinting involves the spatial assembly of different cells, bioinks, and supporting biomaterials, to obtain constructs that mimic the biological and/or mechanical functions of natural tissues (Figure 5).

However, it has been limited by the availability of functional primary human cells and the associated complexity with their use in a clinical setting. In addition, the retention/control of functional cell population within the implanted construct in vivo requires an accurate delivery of regulatory cues (e.g., growth factors) within the printed construct to eventually harness those cells. Engineering cells, through gene delivery, to locally produce those regulatory molecules represents an alternative approach to precisely control cells’ longevity, proliferation, and function within the engineered tissues. The ex vivo cell transfection and their subsequent transplantation in vivo within bioprinted constructs have offered high levels of protein expression over prolonged periods of time. As an alternative, the incorporation of the gene of interest and its delivery carrier into a biodegradable construct, to form a gene activated matrix/bioink, offers a localized and sustained gene delivery to transplanted and host cells, in situ.

In their recent study, Gonzalez-Fernandez T et al. aimed to engineer mechanically functional composites for a range of applications in orthopedic medicine [132]. They have explored the two abovementioned approaches (ex vivo and in situ) to transfect BM-MSCs with genes for osteogenic (BMP2) or chondrogenic (combination of TGF-β3, BMP2, and SOX9) differentiation within networks of 3D-printed thermoplastic fibers. They succeeded to use nanohydroxyapatite particles, and the arginine-alanine-leucine-arginine amphipathic (RALA) peptide as delivery carriers for pDNA to transfect BM-MSCs prior to 3D-Bioprinting and to prepare gene-activated alginate-methylcellulose bioinks.

As mentioned earlier, the delivery of naked pDNA into stem cells without a vector remains a big challenge. Therefore, Nien-Chi Huang et al. have recently developed a rapid and easy method to transfect naked pDNA for GATA-binding protein 4 (GATA4) into human umbilical cord-derived MSCs (hUC-MSCs) by co-extruding naked pDNA and hUC-MSCs with a biodegradable thermo-responsive polyurethane (PU) hydrogel through a micro extrusion-based transient-transfection system [133]. The optimized PU hydrogels induced GATA4-transfected hUC-MSCs to differentiate into cardiomyocyte-like cells within 2 weeks in vitro. Furthermore, they were able to restore the cardiac function in vivo in zebrafish.

## 5. Challenges

Despite the versatile applications of MSCs in biomedicine, many concerns still warrant attention for process optimization such as, timing, dosage, and administration route of transplanted MSCs. For hMSCs to be used in therapy, a large population of hMSCs is needed, thus cells propagated by in vitro expansion. However, the phenotypic and functionality changes following the in vitro expansion of hMSCs are considered a major concern thus far. The effects of replicative/ late-passage senescence on the various functional aspects of MSCs merit further research. In addition, long-term in vitro manipulation or culture of MSCs may lead to genetic instability and various chromosomal abnormalities [134]. This might be due to the diminished efficiency of DNA repair systems, leading to building up of DNA mutations of various types (e.g., deletions, duplications, rearrangements). The in vitro expansion of MSCs is also known to reduce their proliferation and differentiation capacity, bring about cell senescence, and cause various epigenetic changes [135]. Thereafter, younger passages of MSCs are way desirable for transplantation in vivo.

Another factor that impacts MSCs efficacy and/or number is the proper choice of cell source. It is difficult to obtain enough quantities of healthy autologous MSCs with high activity from patients suffering from chronic diseases as diabetes, or rheumatoid arthritis [136]. Moreover, MSCs obtained from subjects with old age or from those with medical conditions are known to hold less therapeutic effects than those obtained from healthy individuals. In such situations, the intrinsic features of MSCs become altered, thus eventually impairing their therapeutic impact. Nevertheless, this concept is opposed by Ivana Ferrero et al. who reported that MSCs obtained from patients with amyotrophic lateral sclerosis (ALS) did not depict any notable functional or chromosomal alterations compared to those obtained from healthy donors [137].

MSCs are famous of their tumor-oriented migration and/or incorporation explaining their use as potential targeted carriers for antitumor drugs/genes. They are attracted to the tumor site by tumor-secreted soluble factors. In addition to their anti-cancer features, MSCs also show both pro-cancer aspects, thus found to be involved in various stages of tumor progression [138]. Upon arrival to tumor, MSCs stimulate epithelial to mesenchymal transition (EMT) of cancer cells. MSCs could also manipulate tumor microenvironment (e.g., by angiogenesis, immunomodulation, etc.,), enhancing tumor progression [138]. Even more, MSCs might enhance the metastatic potential of cancerous cells [139]. On the other side of tumor-MSCs bi-directional interactions, a variety of pro-tumor factors secreted by tumor cells could affect the phenotypic features of MSCs, in addition to their gene expression.

Another major concern with the use of MSCs in cell therapy is their ability to fuse with host cells forming heterokaryons or entosis-derived hybrids with regenerative potential [140]. However, it is still critical to fully investigate the pros and cons of these hybrids. Future studies are still needed to attest how synkaryons would divide and segregate their chromosomes, producing stable/unstable hybrids with regenerative or oncogenic potential, respectively [141]. In case MSCs were genetically modified, the consequences of cell fusion might be even worse, leading to alteration of host cell genome. An attractive approach to guard against damaging consequences of cell fusion is cell entrapment (e.g., microencapsulation, 3D-scaffolds, etc.,). Microencapsulation of human cells has been used for many applications [142,143,144]. In particular, it is extremely useful during cell therapy, wherein the membrane can create a barrier between the host cells and transplanted cells. MSCs could be entrapped within various biomaterials (e.g., hyaluronic acid, alginate, agarose, etc.,) that can also provide a physiologic environment that promotes cell survival [145], functionality [110], and prevent immune response [146] and cell fusion. Even though MSCs are immobilized within the microcapsules, they retain their paracrine functions, and manipulate their microenvironment, at the site of implantation, owing to the expression/overexpression of various therapeutic molecules. Moreover, the microencapsulation per se is considered effective to evade any cell pro-oncogenic risk mediated by direct contact with cancer cells [147].

## 6. Concluding Remarks

Genetically modified MSCs is an attractive tool that could be widely implemented in clinical translation of promising therapeutics. Genetic manipulations in MSCs can be mediated by various delivery systems to efficiently induce the expression, up-regulation, or down-regulation of specific genes or pathways. However, such tool requires detailed investigations on the safety and potency in short- and long-term. In this review we tried to highlight the three main stakeholders of this process, namely the MSCs, nucleic acids, and gene carriers, mainly the non-viral lipid vectors. We tried to emphasize the up-to-date applications for each, as well as the challenges they face. Aiming for optimization of MSCs as a gene delivery carrier, several aspects warrant improvement. The starting point would be by focusing on the biology of MSCs, basically by obtaining cells from a proper source, rightfully characterizing, and carefully expanding them; then, various gene delivery complexes to be optimized for nontoxic transfection greater transgene expression. This entails adequate selection of nucleic acids to match potential applications paying enough attention to their cons and pros. Then, the nucleic acid carriers are optimized by deciding on the suitable biodegradable components and the appropriate preparation method. Moreover, we have highlighted some recent approaches (e.g., bioprinting, bioscaffolds, etc.,) designed to enhance the therapeutic potential of MSCs by augmenting the engrafting niche and/or boosting the targeted delivery for more potent and versatile therapies.

## Figures and Tables

**Table 1:** FDA-approved RNA therapeutics for the treatment of human diseases in chronological order, adapted from [21,22].

Drug Name	Drug Class	Brand Name	Company	Target Disease	Mechanism of Action	Year of Approval	Current Status
Fomivirsen	ASO	Vitravene	Novartis	Cytomegalovirus retinitis	Binds to and blocks translation of IE2 mRNA.	1998	Withdrawn due to decreased need
Pegaptanib	Aptamer	Macugen	OSI Pharmaceuticals	Age-related macular degeneration (wet type)	Binds to and blocks the 165 isoform of VEGF.	2004	Continuous
Mipomersen	ASO	Kynamro	Genzyme Corporation	Homozygous familial hypercholesterolemia	Binds to ApoB mRNA and induces its degradation by RNase H.	2013	Discontinued due to side effects
Nusinersen	ASO	Spinraza	Cold Spring Harbor Laboratory and Ionis Pharmaceuticals	Spinal muscular atrophy	Binds to SMN2 mRNA and alters its splicing.	2016	Continuous
Eteplirsen	ASO	Exondys 51	Sarepta Therapeutics, Inc.	Duchenne muscular dystrophy	Binds to exon 51 and alters splicing of dystrophin pre-mRNA.	2016	Continuous
Patisiran	siRNA	Onpattro	Alnylam Pharmaceuticals Inc.	Polyneuropathy in patients with hereditary transthyretin-mediated amyloidosis.	Binds to transthyretin (TTR) mRNA to decrease hepatic production of TTR protein	2018	Continuous
Inotersen	ASO	Tegsedi	Ionis Pharmaceuticals	Nerve damage in adults with hereditary transthyretin-mediated amyloidosis.	Binds to TTR mRNA and induces its degradation by RNase H	2018	Continuous
Givosiran	siRNA	Givlaari	Alnylam Pharmaceuticals Inc.	Acute hepatic porphyria	Reduces the hepatic production of ALASI protein through interference with ALASI mRNA.	2019	Continuous
Golodirsen	ASO	Vyondys	Sarepta Therapeutics, Inc.	Duchenne muscular dystrophy	Binds to exon 53 of dystrophin pre-mRNA to alter splicing.	2019	Continuous

Note: Antisense oligonucleotides (ASOs), small interfering RNAs (siRNAs).

**Table 2:** A summary of nucleic acids used to transfect MSCs: The advantages and disadvantages.

Nucleic Acid	DNA/RNA	Examples	Pros	Cons	Ref
**Plasmids**	DNA	pCMS-EGFP pUNO1-hBMP-7	Large DNA packaging capacity. Easy to handle. Stable at RT for long periods of time.	Efficient nuclear transport is required. Plasmid backbone elements can induce intracellular inflammation and transgene silencing	[25,57]
**Mini circles**	DNA	McCMV-fLuc2A-EGFP McCMV-CXCR4	High safety profile. Persistent transgene expression (compared to pDNA).	Efficient nuclear transport is required. Sustainable scale up with clinical-grade quality is still needed.	[29,30,31]
**mRNA**	RNA	ΔLNGFR mRNA	No need for nuclear transport. Higher transfection efficiency (compared to pDNA). No risk of genome integration.	Transient expression Repeated dosing required.	[58]
**Oligonucleotides/ ASO**	DNA/RNA	PyNTTTTGT ONs Smurf1 GapmeR	Transient and specific regulation of gene expression. No risk of genome integration	They need delivery carriers. Natural ONs are degraded by nucleases. Binding to off-target RNA. Inability to cross BBB. Could be immunogenic.	[39,40,42]
**Aptamers**	DNA/RNA	HM69 Seq3	High binding affinity to target molecules. Batch-to-batch consistency. Small sizes allowing them to penetrate tissues. Non-immunogenic.	Irrelevant interactions with biomolecules in vivo. Quick excretion via the kidneys.	[46,47]
**RNAi/siRNAs**	RNA	siRNA-Runx2 siRNA–REST TOP2B_5 TOP2B_6	Transient and specific regulation of gene expression. No risk of genome integration.	They need delivery carriers.	[59]
**MiRNAs**	RNA	miR-133 agomir miR-100–5p miR-143–3p	Transient and specific regulation of gene expression. No risk of genome integration.	They need delivery carriers.	[52,53]
**Ribozymes and Deoxy ribozymes**	DNA/RNA	Rzpol1a1	Transient and specific regulation of gene expression. No risk of genome integration.	They need delivery carriers. Off-target effects.	[59,60]
**Short hairpin RNA (shRNA)**	RNA	TIMP-1-shRNA shRNF2-1 shNRF2-2	Specific regulation of gene expression.	Vector-dependent.	[54,55,56]

**Table 3:** Niosomes versus liposomes as gene/drug carriers. [79].

	Niosomes	Liposomes
Components	Surfactant	Phospholipids
Component availability	High	Low
Component purity	Good	Variable
Preparation and storage	No special conditions required	Inert atmosphere and low temperature
Stability	Very good	Low
Cost	Low	High

**Table 4:** Applications of genetically modified MSCs in vivo.

Delivery System	Carrier		Nucleic Acid		Cell Vehicles	Application	Model/Host	Ref
	Type	Composition	Vector	Delivered Gene/siRNA				
**Non-viral**	Liposomes	Lipofectamine Plus^®^	Plasmid DNA	hTERT	MSC line derived from fetal porcine pancreas	Hyperglycemia	Diabetic model/Kunbai strain mice	[113]
	Polymer	PEI	Plasmid DNA	TRAIL	BM-MSCs	Melanoma	Melanoma model/e C57BL/6 mice	[115]
	Polymer	Chitosan	Plasmid DNA	BMP-2	BM-MSCs	Bone regeneration	Calvarial defect model/Rats	[85]
	Polymer	PEI	Plasmid DNA	BMP-2	BM-MSCs deriver MVs within gene-activated scaffold (DBM/MVs-PEI/phBMP2)	Bone regeneration	Femoral condylar defect/New Zealand white rabbits	[86]
	Polymer	Alginate GAM	Plasmid DNA	BMP-2	Rat BM-MSCs	Bone regeneration	Orthotopic spinous process defect/Fischer 344 inbred rats	[87]
	Polymer	LPEI	Plasmid DNA	VEGF	BM-MSCs	Myocardial infarction	MI model/SD rats	[95]
	Polymer	Cationized pullulan	Plasmid DNA	Suicide gene (CMV-TK)	Rat BM-MSCs	Melanoma	Pulmonary melanoma metastasis model/C57BL6 mice	[83]
	Polymer	LPEI	Plasmid DNA	CDY::UPRT	AT-MSCs	GDEPT: Chemo-resistant glioblastoma	Temozolomide resistant U-251MG cells/Nude mice	[116]
	Polymers	PEI-PLGA	Plasmid DNA and siRNA	coSOX9-pDNA/Cbfa-1-siRNA	hMSCs encapsulated in fibrin hydrogels	Chondrogenic differentiation	Nude BALB/c mice	[117]
	Polymers	PLL-PEI	Plasmid DNA	HSV-TK and TRAIL	rMSCs	Glioblastoma	Glioma model/SD rats	[101]
	Polymeric NPs	BA-PEI	Plasmid DNA	VEGF	BM-MSCs	Myocardial infarction	MI model/SD rats	[96]
	Plasmid-activated scaffolds	Chitosan-gelatin andnHA	Plasmid DNA	TGF-β1 and BMP-2	BM-MSCs	Regeneration of articular cartilage and subchondral bone	Knee ostochondral defect model/Rabbits	[118]
	nHA dual gene-activated scaffold	nHA and PEI	Plasmid DNA	BMP-2 and VEGF	rMSCs	Bone regeneration	Critical-sized cranial bone defect model/Rats	[88]
	Peptide conjugated NPs	Cationic AuNPs and PEP	Plasmid DNA	VEGF	Rat BM-MSCs	Antimicrobial and wound healing properties	Infected full thickness skin defect model/Mice	[114]
**Viral**	AAV		IL-10		hBM-MSCs	Cerebral ischemia	MCAO I/R model/SD rats	[82]
	Adenovirus		HSV-TK/GCV		BM-MSCs	Intracranial gliomas	Intracranial human U87 glioma model/Nude mice	[100]
	Adenovirus		HGF		hBM-MSCs	Spinal cord injury	Spinal cord injury model/ SD rats	[80]
	Adenovirus		EGFR		Murine BM-MSCs	Brain tumors	Intracranial GL261 glioma or B16 melanoma/C57BL/6 mice	[119]
	Adenovirus		IFN-β		hBM-MSCs	Pancreatic cancer	Transplant PANC-1 cancer model/SCID mice	[103]
	Fiber-modified adenovirus		kringle1-5/EGFP		hPMSCs in Matrigel plugs	Suppression of angiogenesis	Subcutaneous cell loaded matrigel plugs/ BALB/c nude	[105]
	Gamma -Retrovirus		IL7-IL12		hBM-MSCs	Colorectal cancer	Transplant LS174T colorectal cancer model/NSG mice	[102]
	Gamma-retrovirus		HSV-TK		hBM-MSCs	Gastrointestinal/ hepatopancreatobiliary adenocarcinoma	Phase I and II clinical trial	[84]
	HSV-1		HGF		rBM-MSCs	Cerebral ischemia	MCAO I/R model/Wistar rats	[81]
	Lentivirus		miR-126		BM-MSCs	Myocardial infarction	MI model/Mice	[99]
	Lentivirus		HGF		UCB-MSCs	Myocardial infarction	MI model/SCID mice	[97]
	Lentivirus		FGF21		Mouse BM-MSCs	Brain Injury	Impact-induced traumatic brain Injury model/C57BL/6 mice	[92]
	Lentivirus		CXCR4		rBM-MSCs	Cerebral ischemia	MCAO I/R model/SD rats	[94]
	Recombinant adenovirus		VEGF		BM-MSCs	Cerebral ischemia	MCAO I/R model/rats	[93]
	Retrovirus		AKT		Mouse BM-MSCs	Myocardial infarction	MI model/C57BL/6 mice	[98]
**Hybrid**	Adenovirus/liposome		Ad-hEndo		hPMSCs	Ovarian cancer	Transplant A2780 ovarian cancer model/ Nude mice	[104]
	Adenovirus/CPP		stTRAIL		hUCB-MSCs	Glioblastoma	Intracranial xenograft human glioma model/Athymic nude mice	[120]
	Adenovirus/4HP4		IL-12M		rBM-MSCs	Melanoma and cervical cancer	B16F10 melanoma and TC-1 cervical cancer models/SCID mice	[121]

Note: 4HP4: tetrameric form of cell-permeable peptide; CPP: cell-permeable peptide; HSV: herpes simplex virus; tTATop-BMP-2: tetracycline transactivator and BMP-2 cDNAs; BA-PEI: bile acid-modified polyethyleneimine; PMAA: polymethacrylate acid; CMV: cytomegalovirus; AT-MSCs: adipose tissue-derived MSCs; HIF-1 α: hypoxia-inducible factor-1α; CDY::UPRT: cytosine deaminase and uracil phosphoribosyl transferase; GAM: gene-activated matrix; GDEPT: gene-directed enzyme prodrug therapy; MVs: microvesicles; DBM: demineralized bone matrix; PLL-PEI: polylysine-modified polyethylenimine; hPMSCs: human placenta-derived MSCs; HSV: herpes simplex virus; MCAO I/R: middle cerebral artery occlusion ischemia/reperfusion; SD: Sprague-Dawley; MI: myocardial infarction; hEndo: human endostatin; UCB-MSCs: umbilical cord blood-derived MSCs.

## Data Availability

Not applicable.
